# Microbiomes in the context of developing sustainable intensified aquaculture

**DOI:** 10.3389/fmicb.2023.1200997

**Published:** 2023-06-23

**Authors:** Marlene Lorgen-Ritchie, Tamsyn Uren Webster, Jamie McMurtrie, David Bass, Charles R. Tyler, Andrew Rowley, Samuel A. M. Martin

**Affiliations:** ^1^School of Biological Sciences, University of Aberdeen, Aberdeen, United Kingdom; ^2^Centre for Sustainable Aquatic Research, Swansea University, Swansea, United Kingdom; ^3^College of Life and Environmental Sciences, University of Exeter, Exeter, United Kingdom; ^4^Centre for Environment, Fisheries and Aquaculture Science (Cefas), Weymouth, United Kingdom; ^5^Department of Biosciences, Faculty of Science and Engineering, Swansea University, Swansea, United Kingdom

**Keywords:** functionality, health, immune system, microbiota, sustainability

## Abstract

With an ever-growing human population, the need for sustainable production of nutritional food sources has never been greater. Aquaculture is a key industry engaged in active development to increase production in line with this need while remaining sustainable in terms of environmental impact and promoting good welfare and health in farmed species. Microbiomes fundamentally underpin animal health, being a key part of their digestive, metabolic and defense systems, in the latter case protecting against opportunistic pathogens in the environment. The potential to manipulate the microbiome to the advantage of enhancing health, welfare and production is an intriguing prospect that has gained considerable traction in recent years. In this review we first set out what is known about the role of the microbiome in aquaculture production systems across the phylogenetic spectrum of cultured animals, from invertebrates to finfish. With a view to reducing environmental footprint and tightening biological and physical control, investment in “closed” aquaculture systems is on the rise, but little is known about how the microbial systems of these closed systems affect the health of cultured organisms. Through comparisons of the microbiomes and their dynamics across phylogenetically distinct animals and different aquaculture systems, we focus on microbial communities in terms of their functionality in order to identify what features within these microbiomes need to be harnessed for optimizing healthy intensified production in support of a sustainable future for aquaculture.

## 1. Introduction

As the global population continues to expand rapidly, so too does the need for sustainably produced food. The world’s water bodies cover over 70% of the planet and are home to a bounty of protein-rich seafood (edible fish, invertebrates and algae from marine, brackish and freshwater environments). Global consumption of seafood (from capture and culture production in inland and marine waters) is growing at an annual rate of 3.1% (1961–2017), which is faster than for any livestock and animal production sector (FAO, 2020). Concurrent with stagnation of wild fisheries, aquaculture has progressively increased its contribution to fish and shellfish production with a mean annual growth of 5.3% between 2001 and 2018 (FAO, 2020), accounting for 52% of all seafood produced for human consumption in 2018 (FAO, 2020). The continued expansion of aquaculture has however been hampered by outbreaks of infectious disease, concerns over environmental footprint, and impacts of climate change.

Health management in aquaculture has historically relied upon antibiotics and other chemotherapeutics (de Bruijn et al., 2018), but continued development of targeted vaccines (Ma et al., 2019) and selective breeding to generate disease resistant lines (Houston et al., 2020) are now coming to the forefront as environmentally sustainable alternatives. A more holistic approach to health management in aquaculture is to consider the cultured animal and its surrounding environment together. Emerging evidence indicates that microbial communities both within and surrounding an animal in aquaculture can contribute directly to productivity in terms of growth, disease resistance and animal welfare (Perry et al., 2020; Rajeev et al., 2021; Dai et al., 2022; Murphy et al., 2022). Microbial communities play dynamic functional roles with both commonalities and contrasts across cultured animal groups from invertebrates such as mollusks and crustaceans to vertebrates—predominantly finfish—and should be viewed as central components in the development of a sustainable aquaculture industry.

**Figure 1:**
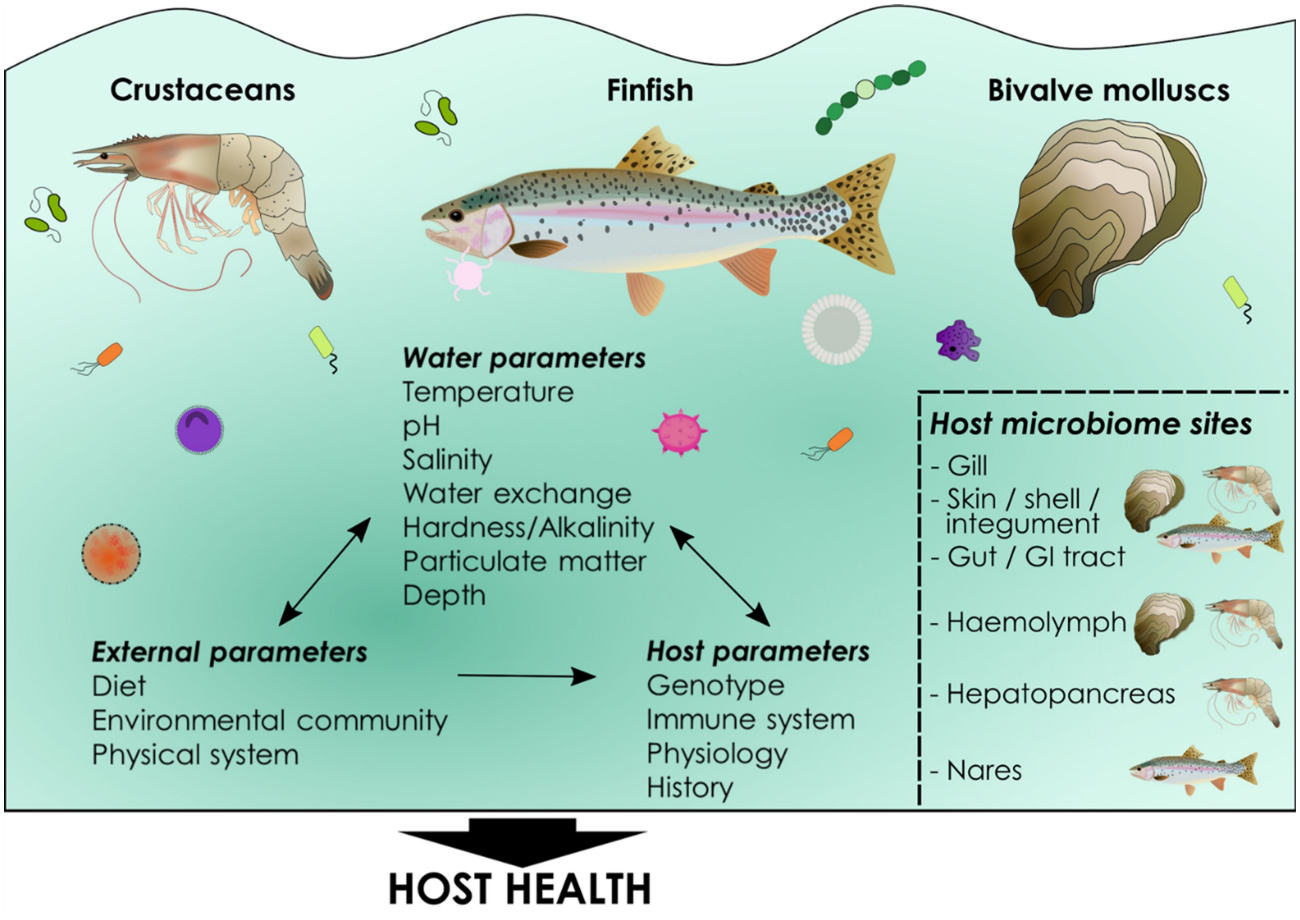
What is a microbiome? A microbiome can be defined simply as “a community of commensal, symbiotic, and pathogenic microorganisms within a body space or other environment” (Lederberg and McCray, 2001), although many varying definitions exist (Berg et al., 2020). A microbiome comprises a community of microbes including bacteria, viruses, fungi, microeukaryotic and metazoan parasites and Archaea (collectively known as the microbiota) as well as the downstream products and functionality of the microbiota. Cultured aquatic species harbor communities of microbes in the gut or gastrointestinal tract, as well as in the skin, nasal and oral cavities, gills, hemolymph, shells, integument and hepatopancreas, some of which are common across all cultured animal phyla (e.g., gills). The established microbiome composition is complex and involves not only host factors such as genotype and the host immune system, but also external environmental influences such as diet, physical parameters of culture systems, microbes in the aquatic environment and an array of water physiochemical parameters, including temperature, salinity, and dissolved oxygen. Together these parameters determine microbiome composition in host niches and these communities of commensals influence host health. Key determinants of host microbiome composition in cultured aquatic animals. Host microbiomes are determined by both environmental and host-associated factors. Environmental parameters can also reciprocally impact each other, as well as the host, particularly the microbial community within the water. Environmental parameters can be measured and, in some cases, controlled in aquaculture as can host factors such as genetics. Microbiomes occur in all cultured taxa including finfish, bivalve mollusks and crustaceans. Common microbial niches are found in a number of mucosal tissues including gut, gill and skin while others are specific to particular phyla, for example invertebrate hemolymph. Host microbiome composition itself has important implications for host health, but the functional and mechanistic pathways underlying these associations are poorly understood in aquaculture.

The associated microbial communities both within and surrounding farmed aquatic animals are commonly referred to as the microbiome (Figure 1). Bacterial communities are the focal point of the vast majority of microbiome studies and a paucity of information exists relating to the communities of other microbes including viruses, fungi, archaea and protozoa. The fungal community has been characterized in telelosts including zebrafish, the gut of which contained fungal taxa belonging to more than 15 classes across the phyla *Ascomycota*, *Basidiomycota*, and *Zygomycota*, and distinct profiles in wild versus laboratory reared fish (Siriyappagouder et al., 2018). In the wood-eating Amazonian catfish (*Panaque nigrolineatus*), the mycobiome varied in different gut regions, and as a function of diet (Marden et al., 2017). Using a metatranscriptomic approach in the intestine of gilthead sea bream (*Sparus aurata*), fungal transcripts were found to be as abundant as bacterial, and while bacteria were important in vitamin and amino acid metabolism as well as rhythmic and symbiotic pathways, fungi were determined to play a role in host immunity, digestion and endocrine processes (Naya-Català et al., 2022). In green-lipped mussels (*Perna canaliculus*), the fungal community was determined in gills, hemolymph, digestive gland and stomach, and the majority of amplicon sequence variants (ASVs) belonged to the phylum *Ascomycota* in line with other studies (Li et al., 2022). However, more than 50% of ASVs for fungal taxa were only classified at the phylum level, highlighting the lack of fungal studies and thus lack of reference sequences, a hurdle which will need to be overcome to fully characterize complete microbial communities.

Across cultured phyla there are common tissues which host microbial communities including gut and gill, but microbiomes also occur in fish on their skin, and in their nasal and oral cavities, and in crustaceans and mollusks in their hemolymph (blood), digestive glands, shells (mollusks) and integument (crustaceans). Differences in microbiome composition in different tissues is driven by niche specialization and host selective pressures (Kelly and Salinas, 2017). A common feature across phylogenetically distinct animals in aquaculture is the primary role of internal mucosal microbiomes in digestion and nutrient absorption while external mucosal communities assume a primarily defensive role from the perspective of outcomes for the host. Yet microbiomes can also contain opportunistic pathogens, and to some extent are determined by the surrounding environment and not regulated by the host. In this perspective we explore how microbiomes contribute to productivity and health in aquaculture, with a particular focus on the commonalities and differences between phylogenetically diverse cultured animals and culture systems. We also present future prospects on how we may harness the power of the microbiome to promote sustainable aquaculture as production seeks to be intensified.

## 2. Establishing the host microbiome—where do commensal microbes come from in aquaculture?

Distinct from their terrestrial counterparts, aquatic organisms are in constant contact with (potentially) microbially rich water and this constant exposure may offer adaptive advantages, in addition to some inherent threats. In aquaculture systems, the aquatic environment is highly dynamic and can change both frequently and rapidly in response to many factors, including weather, temperature, and husbandry practices. The majority of aquaculture species, both invertebrate and vertebrate, reproduce by external fertilization, producing an independent egg exposed to the environment. External mucosal surfaces are first colonized by environmental microbes shortly after hatching (Giatsis et al., 2014; Stephens et al., 2016). As initial colonizers originate from the environment, hatchery conditions play a strong role in determining the diversity and richness of the initial colonizing community (Minich et al., 2021). In contrast, the internal gut mucosa is not colonized until the time of mouth opening and first feeding, exposing this internal niche to a potentially different pool of colonizers from the environment as well as diet-associated microbes. Critically, a sterile or partially sanitized environment restricts the pool of microbes from which to establish resident communities.

### 2.1. Selectivity in colonization—how to tell friend from foe?

Interactions between mucosal microbiota and the host immune system is particularly intense during initial colonization by commensals. Commonalities exist between invertebrates and vertebrates during the first phase of colonization in that acceptance or rejection of microbial taxa is controlled by a triad of the environmental conditions, the microbes themselves, and the host innate immune system. Germline-encoded cell receptors, where they exist, can play an early role in community selection and the innate immune system must have the ability for tolerance and allow settlement of commensals while selectively excluding opportunists or undesirable taxa (Kelly and Salinas, 2017). These cell receptors are likely to differ between mucosal surfaces, which raises interesting mechanistic questions. However, it is not solely the case that the immune system “selects” symbionts, but also that microbes from the environment may successfully colonize as they are best adapted to survive in the nutrient rich biochemical environment of the mucosal surface, independent of host influences.

In contrast with crustaceans and mollusks, the immune systems of which are devoid of lymphocytes and immunoglobulins (Igs), the acquired immune system or immunological memory capability of fish develops at first-feeding (Castro et al., 2015), while initial colonization of the gut occurs between mouth opening and first-feeding. As such, in addition to being exposed to a temporally distinct group of potential colonizers, colonization of the gut may be regulated by different immune pathways in invertebrates and vertebrates. In fish, as in other vertebrates, Igs are a key feature in acquired mucosal immunity, coating resident microbes to aid in selectivity (Woof and Mestecky, 2005; Kelly and Salinas, 2017). Dominant Ig isotypes have been identified at different mucosal sites, suggesting a role in determining site specific microbiota (Salinas et al., 2021). Although mollusks and crustaceans lack immunological memory in terms of Ig-mediated mechanisms, they harbor complex repertoires of lectins and other binding molecules which enable recognition of diverse microbes, resulting in control of undesirable species for successful innate immune protection (Vasta et al., 2007, 2012; Gerdol et al., 2018; Coates et al., 2022). Thus, successful colonizers are microbes in the environment which are suited to a mucosal niche and evade exclusion by the host immune system. This has been well studied in bobtail squid, *Euprymna scolopes* which acquire a specific strain of *Vibrio fischeri* from the surrounding environment into a specialized light organ, excluding all other bacterial taxa (Kostic et al., 2013). An additional facet of the immune system to consider when trying to understand microbial colonization are antimicrobial peptides (AMPs). AMPs refer to small peptides also known as bacteriocins including hepcidins, beta-defensins, cathelicidins and fish-specific piscidins (Masso-Silva and Diamond, 2014; Katzenback, 2015). AMPs are generally toxic to closely-related strains, although wider target ranges also occur (Rodney et al., 2014) and thus have the potential to impact colonization and succession by making an inhospitable environment for certain taxa. Less well understood in aquaculture is the interaction between environmental microbes and host genetics in microbiome colonization.

### 2.2. How do host genetics impact microbial colonization?

Host genetics may play a role in the establishment of microbial associations through microbial recognition, immune selection, and determination of the biochemical niche (Spor et al., 2011). Fish of different species reared in the same or closely related environments can differ in their microbiome composition (Nikouli et al., 2021). Additionally, a genetic component may persist within species. In genetically divergent three-spined stickleback (*Gasterosteus aculeatus*) populations, divergence in the intestinal microbiota is greater than could be accounted for by environment and ecology, supporting a role for host genetics in the selection of bacterial species (Smith et al., 2015; Steury et al., 2019). Even within populations, an interaction between major histocompatibility complex class IIb (MHC class IIb) polymorphisms and genotype could impact microbiome diversity (Bolnick et al., 2014). However, other studies in Atlantic salmon (*Salmo salar*) identified a relatively minor role of host genetics (Dvergedal et al., 2020), particularly when compared to environmental effects (Uren Webster et al., 2018). Yet using hybrid populations of Chinook salmon, compositional differences in microbiota were attributed to quantitative genetic architecture (Ziab et al., 2023). It may be the case that, in common with mammalian systems, genotype-dependent selection may influence specific microbes, as opposed to the whole microbial community (Tabrett and Horton, 2020). Some animals in aquaculture, particularly salmonids, generally originate from brood-stock under genetic selection for production traits, notably related to growth, which may influence the contribution of environmental relative to genetic factors. In the case of shrimp farming, traits selected for are almost entirely focused on disease resistance. Importantly, genetic processes select microbes after the host has come into contact with bacterial communities in the environment, an important consideration in trait selection for different environments.

Little is known about the interactions between host genetics, microbiome stability, and health outcomes in relation to animals in aquaculture. Pacific oyster [*Crassostrea* (*Magallana*) *gigas*] families susceptible to Ostreid herpesvirus-1 have been shown to have differences in bacterial community structure and evenness compared to resistant families (King et al., 2019; Clerissi et al., 2020) and there are similar observations for the gill and gut of susceptible and resistant rainbow trout (*Oncorhynchus mykiss*) in response to *Flavobacterium psychrophilum* infection (Brown et al., 2019). In addition to genetic influences, epigenetic processes provide a link between host and microbiota. Metabolites resulting from cellular metabolic processes can impact the activity of enzymes involved in histone and DNA methylation and demethylation (Narne et al., 2017; Reid et al., 2017). The environmental microbiome is important in host immune system maturation, including the development of pathogen recognition systems. In the Pacific oyster, early life exposure to microbes improved survival during a challenge with Pacific oyster mortality syndrome (POMS) not just in the exposed generation, but in the following generation also. A combination of microbiota, transcriptomics, genetics and epigenetic analyses determined a distinct change in epigenetic methylation marks during microbial exposure leading to an altered immune gene expression and long-lasting, intergenerational immune protection against POMS (Fallet et al., 2022).

Overall, host genetics appear to play a central role in microbiome establishment, but little is known about the genomics *x* environment interaction for establishment and maintenance of microbial communities. The interactive nature of genetics and environment raises the potential for targeted environmental conditioning in aquaculture. To consider such approaches, it is important to first understand the role of physical aquaculture systems in dictating parameters which influence both host and environmental microbes.

## 3. Aquaculture system dynamics and host microbiome determination

Water has many variable properties including temperature, salinity, pH, ionic composition and chemistry related to dissolved gasses. Additionally, physical aquaculture systems dictate water exchange rate, retention time, and a level of biological sterility, particularly in hatcheries, a key stage for community establishment. Water harbors an array of microbes which provide ecosystem services within wild, and aquaculture systems and system parameters are important determinants of microbial dynamics. In low diversity microbial environments with input of organic material, fast-growing opportunistic *r*-strategists characteristic of instability can outcompete slow-growing *K*-strategists which are markers of stability. However, if water retention time in the system is short, opportunists are less able to replicate and stability of the system is more likely maintained by virtue of this management practice (Attramadal et al., 2012; Vadstein et al., 2018). A key end-goal of system optimization in aquaculture is to define environmental parameters which are conducive to desirable microbiomes in terms of sustainability and productivity, and to then have the ability to monitor and control these parameters to ensure optimum performance. However, due to the wide range of aquaculture system types (categorized as flow-through, open, or recirculating) and their associated temporal dynamics, a “one-size fits all” approach to determining optimum parameters is unlikely to succeed.

### 3.1. Aquaculture systems and evolving microbial stability

**Figure 2:**
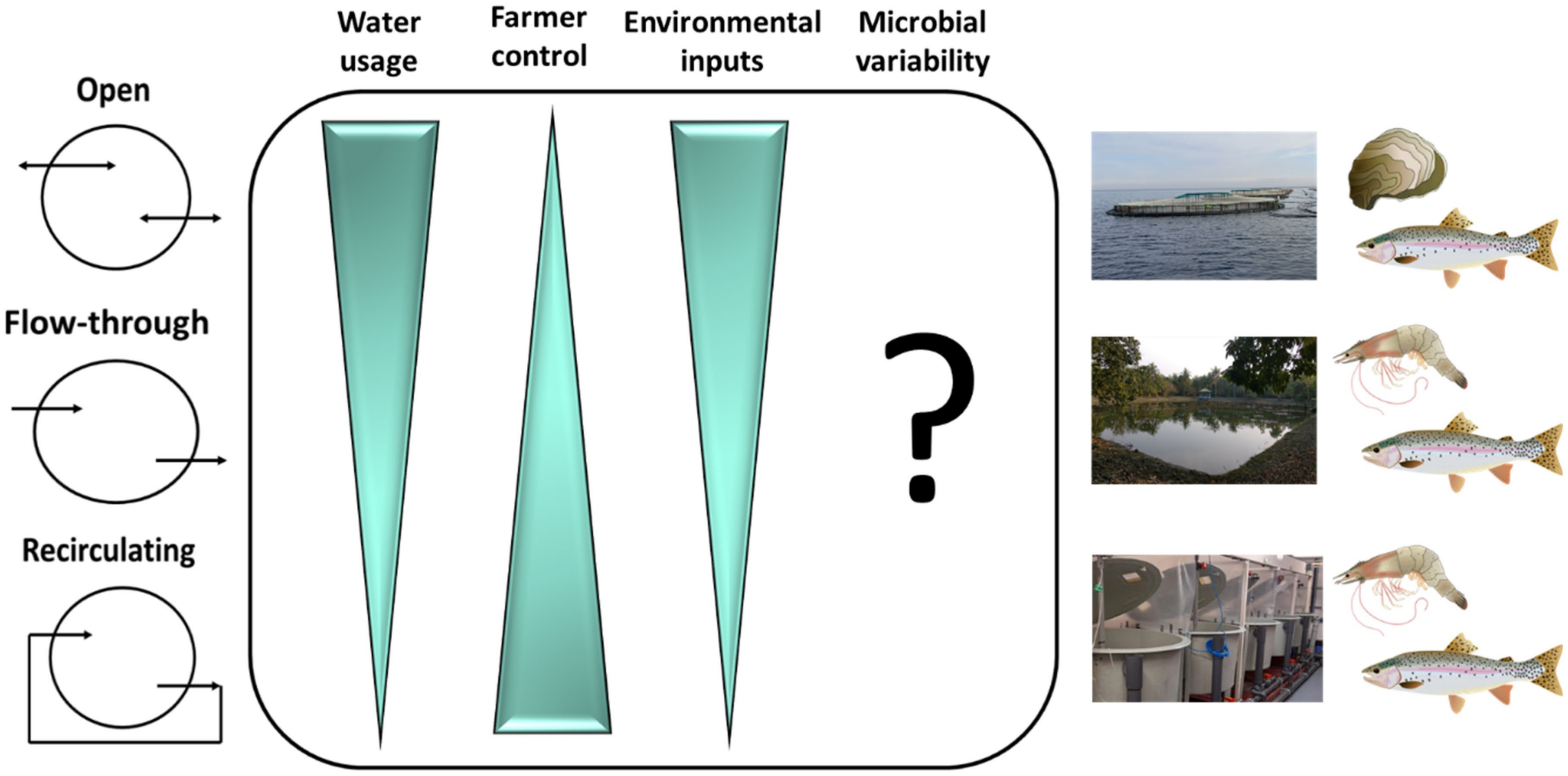
Microbiome dynamics in different aquaculture systems. Water usage, farmer control, environmental inputs and microbial variability in aquaculture systems. An important consideration in microbial maturation within aquaculture systems is water usage, particularly in terms of water flow and exchange. Systems vary from open (e.g., Atlantic salmon pens in lochs or fjords) to closed (e.g., land-based recirculating aquaculture systems; RAS) with systems between the two extremes referred to as flow-through (e.g., tilapia or shrimp ponds). Farmer control over environmental parameters including temperature and photoperiod are greater moving from open to recirculating systems and environmental factors such as natural seasonality and exposure to pollutants decrease. These dynamics may suggest a more stable microbial environment in closed systems, but in reality microbial variability is unlikely to follow a gradient from open to closed systems. Open systems are exposed to a rich pool of natural microbes which vary with season and geographic location. Contrastingly, strict husbandry practices to ensure pathogen exclusion in RAS, may result in periods of near sterility in the system, effectively re-setting the microbial community and introducing variability. In both cases, the result is potential instability in the system, but it is not known if stability is essential to a functioning microbiome.

The nature of the aquaculture system can have major impacts on microbiomes of finfish (Minich et al., 2020, 2021; Uren Webster et al., 2020) and invertebrates, as illustrated for oyster (Arfken et al., 2021) and shrimp (Tepaamorndech et al., 2020). Such impacts are determined by physical and environmental parameters within systems and can influence production outcomes including growth and survival (Table 1). Culture systems come in an array of shapes and sizes and can be broadly described on a scale from open to closed (Figure 2). Larval stages are generally cultivated in highly controlled flow-through or recirculating aquaculture system (RAS) hatcheries with many species requiring planktonic organisms as feed before being transferred to on-growing facilities that vary between species. On-growing occurs in tanks, ponds and cages, or on other structures such longlines and rafts for bivalves (Tidwell, 2012). Most commonly these on-growing stages are exposed to the natural environment and generally employ the use of processed diets for finfish and crustaceans, or natural feed for filter feeding animals. Most open systems such as sea cages are exposed to natural fluctuations in the environment and may experience temperature extremes, exposure to harmful algal blooms, jellyfish (Clinton et al., 2021), and pollutants (Trabal Fernández et al., 2014). Such challenges have direct impacts on microbial communities and exposure to pathogens. The desire to have greater control over environmental parameters with a view to optimizing water quality to promote growth and minimize pathogen exposure, has led to an expansion of closed or semi-closed systems such as RAS. However, although it is more feasible in closed environments to maintain a high degree of stability, water chemistry can change over time in closed systems, depending on how they are maintained, and this can affect the biological load (Fossmark et al., 2020) and in turn the environmental microbiome. How the physiochemistry of the different aquaculture systems may affect microbial stability in aquaculture is now a significant area of research interest. Although the physical systems in which invertebrates and vertebrates are reared in and on-grown differ, commonalities can be defined in terms of the nature of the systems on the scale from open to closed.

**Table 1:** Key published literature describing system parameter effects on animal microbiomes and production outcomes.

Species	Microbiomes (s)	System (s)	Factor	Description	References
Atlantic salmon (*Salmo salar*)	Gut and water	RAS	Membrane filtration	Salmon parr in RAS with membrane ultrafiltration (mRAS) compared against conventional RAS (cRAS) in periods of high and low organic loading. With high organic loading in cRAS, opportunistic bacteria colonized the gut microbiome. Ultrafiltration in mRAS stabilized the water microbiota, preventing growth of and colonization by opportunists.	Bugten et al. (2022)
Nile tilapia (*Oreochromis niloticus*)	Gut	RAS and FTS	Hatchery type	Tilapia embryos reared to fry in FTS, RAS or RAS with probiotic feed (RASB). Lower survival in fish reared in FTS compared to RAS and RASB. Gut microbiota composition differed in fish from different hatchery types and showed correlation with survival rate and size at harvest.	Deng et al. (2022)
Tilapia (*Coptodon rendalli* and *Oreochromis shiranus*)	Skin and water	Pond	Location	Tilapia reared in seven earthen ponds in two pond systems in distinct geographic regions. 92% of taxa shared by skin and water, but enriched and core taxa differed. Strong site-specific clustering of water samples, but not skin, highlighting some independence of skin microbiome from that of the environment.	McMurtrie et al. (2022)
Common carp (*Cyprinus carpio*)	Gut, sediment and water	Pond	Water quality	Carp reared in pond and sampled on 10 occasions across five months. The impact of water quality on water microbiota was stronger than the influence of gut or sediment microbiota. Gut microbiota dynamics were most closely associated with sediment microbiota.	Jing et al. (2021)
European lobster (*Homarus Gammarus*)	Larvae, biofilm and water	RAS and FTS	UV disinfection	Lobster larvae reared in RAS, RAS with UV disinfection or FTS in two separate experiments. Significantly different larval and water microbiomes were identified in each system. Survival was consistently highest in RAS without disinfection in replicate tanks and experiments.	Attramadal et al. (2021)
Eastern oyster (*Crassostrea virginica*)	Larvae and water	RAS and FTS	Hatchery type	Compared microbiomes of larvae originating from 4 different hatcheries for two consecutive spawning events. Larval microbiota were distinct from water and between hatcheries and spawning events. Hatchery had the strongest effect. Core OTUs (n = 25) identified across larval microbiomes.	Arfken et al. (2021)
Nile tilapia (*Oreochromis niloticus*)	Gut, biofloc, water and feed	RAS	Biofloc	Tilapia reared in RAS, RAS with *in situ* biofloc or fed a diet containing live or dead *ex situ* biofloc. *In situ* biofloc increased microbiome diversity in the gut with an increase in abundance of potentially beneficial taxa. Growth was also increased in fish from the *in situ* biofloc treatment.	Deng et al. (2021)
Pacific whiteleg shrimp (*Litopenaeus vannamei*)	Gut and biofloc	Static tank	Biofloc	Shrimp reared in indoor tanks with no water exchange, with or without biofloc. Microbiota composition similar with or without biofloc, but individual taxa enriched. Expression of immune-related genes and immune status enhanced in shrimp reared with biofloc.	Tepaamorndech et al. (2020)

RAS, recirculating aquaculture system; FTS, flow-through system.

### 3.2. Open versus closed—what do aquaculture system structures mean for the microbiome?

In open aquaculture systems with constant water exchange—such as those used in shellfish cultivation and finfish cage culture—water quality, chemistry and microbial composition vary with geographical location, time, and season, with temperature having a strong impact on the microbiota (Sunagawa et al., 2015). For example, in response to increased water temperature, a surge in opportunistic pathogenic taxa in the Pacific oyster, *C. gigas* with dominance of *Vibrionaceae* occurs (Green et al., 2019). Similarly in marine Atlantic salmon, gut microbiomes are dominated by *Leuconostoc* and *Weissella* in cold water temperatures, but by *Vibrio*, *Allivibrio*, and *Photobacterium* in warmer water (Zarkasi et al., 2014). Circadian rhythmicity is naturally determined by latitude and influences microbiome composition in rainbow trout skin (Ellison et al., 2021), but photoperiod is also often manipulated in aquaculture to promote growth and enable year-round production (Mero et al., 2019; Wang et al., 2020; Pino Martinez et al., 2021), and is likely to impact on immune function with consequences in mucosal immune activity (West et al., 2021).

In RAS, microbial processes are vital in maintaining water quality (Attramadal et al., 2012; Blancheton et al., 2013). Biofilters in the RAS loop host essential communities of bacteria which regulate conversion of waste nutrients to prevent build-up of toxic metabolites such as ammonia and hydrogen sulfide resulting from uneaten feed and fecal matter. Build-up of organic matter or introduction of pathogens result in mass mortalities, and therefore various forms and combinations of filtration and disinfection are often carried out in a RAS loop (Summerfelt et al., 2009). Filtration in the RAS loop assists in stabilizing carrying capacity (Fossmark et al., 2020), while disinfection is an effective means of removing pathogens, but may effectively “re-set” the community. Such dynamics raise questions about how, where, and when RAS water should be subject to disinfection. Simply put, how clean is too clean? At the other end of the sterility scale, the use of biofloc technology, a microbial feed source added to culture water to maintain microbial communities in culture water, has associated benefits for immune reactivity in shrimp (Tepaamorndech et al., 2020), and faster growth in tilapia (Nguyen et al., 2021). Importantly, despite the relative stability of RAS in comparison to open systems, microbiomes in Atlantic salmon gut in RAS are still temporally dynamic, possibly related to biological load in the system (Lorgen-Ritchie et al., 2021).

Intermediate between open and closed states, pond systems are the dominant forms of global aquaculture, particularly prominent in low-middle income countries of Asia, responsible for farming carp, tilapia and shrimp (FAO, 2020). Similar to open systems, ponds are exposed to fluctuating external environmental parameters, but the physiochemical and biotic condition of ponds can shift rapidly due to their shallow depth and limited water exchange, especially when compared to the large water body buffer of open cage culture. During the rainy season, rapid salinity fluxes occur due to rainwater diluting ion concentrations. In intensive culture, algal blooms can form due to excessive food and fertilizer (e.g., manure) inputs providing the key phytoplankton nutrients phosphorous and nitrogen (Huang et al., 2016). In summer months overgrowth of photoautotrophic bacteria can lead to highly turbid pond water, limiting oxygen production (Sriyasak et al., 2015; Dien et al., 2019). In the oriental river prawn (*Macrobrachium nipponense*) hypoxia induced changes in the intestinal microbiome composition with a deviation toward higher levels of pathogenic bacteria (*Aeromonas*) (Sun et al., 2020). Mitigation strategies for anoxic conditions include pond aeration (Boyd, 1998), and multi-trophic farming with mollusks that filter feed on phytoplankton resulting in a net increase of dissolved oxygen (Cunha et al., 2019). As aquaculture continues to expand, it would be beneficial to employ an ecosystem-based approach when designing and optimizing aquaculture systems, attempting to integrate interactions between physical environment, host, and microbiome to promote establishment of desirable microbiomes for productivity and host health.

## 4. Which microbiomes promote animal health in aquaculture?

Productivity and welfare in all areas of the aquaculture industry are under constant threat from opportunistic pathogens, infectious disease and syndromes which result in poor health. A growing body of evidence challenges the idea of a single pathogen causing a single disease; the concept of a “pathobiome” implies that a triad of interactions between environmental microbes, host-associated commensals and the host itself steer overall host health (Bass et al., 2019). For example, in skin mucus of brook charr (*Salvelinus fontinalis*), host stress was associated with a decreased abundance of beneficial commensals including *Methylobacterium* and *Propionibacterium*, known to produce pathogen growth inhibitors poly-β-hydroxybutyrate and bacteriocin, coincident with a rise in abundance of opportunistic pathogenic genera including *Aeromonas*, *Psychrobacter* and *Acientobacter* (Boutin et al., 2013). Additionally, cryptic infection is possible whereby pathogens with dormant, or non-pathogenic life stages may infect a host asymptomatically, but be activated as a pathogen in response to a change in the environment, for example, seasonal changes in temperature (Overstreet and Lotz, 2016; Bass et al., 2019). Mucosal surfaces and their associated microbiota are the first line of defense against pathogens and are equipped with a range of protective molecules such as antimicrobial peptides and immune-related cell components (Reverter et al., 2018) which may induce competitive advantage directly or indirectly. It is becoming increasingly clear that disease impacts host microbiomes, but also that host micorbiomes can mediate disease severity or progression (Table 2). For example, in Atlantic salmon infected with amoebic gill disease (AGD), a significant reduction in bacterial diversity was apparent when compared with healthy individuals and a particular taxon (*Tenacibaculum dicentrarchi*) was particularly prevalent in lesions of infected gill tissue (Slinger et al., 2020). In red abalone suffering from Withering syndrome (WS), *Mycoplasma* was replaced as the dominant taxon in the digestive gland by *Candidatus Xenohaliotis californiensis*, but *C. X. californiensis* was also a member of the microbiome of healthy individuals, suggesting that in this case, the ratio between the two taxa may be key in determining pathogenicity of the disease (Villasante et al., 2020). The array of life history strategies, species and systems across which aquatic animals are farmed represent a challenge in identifying desirable microbiome signatures at the taxonomic level, yet at a functional level, commonalities can be identified. In this line of thinking, it is the ability of the microbiome to maintain functionality in the face of environmental change that promotes host health, rather than consistency of the taxonomic composition of microbial communities.

**Table 2:** Key published literature exploring the interaction between disease and host microbiome composition.

Species	Tissue (s)	Disease	Description	References
Crayfish (*Procambarus clakii*)	Gut and hepatopancreas	White spot syndrome virus (WSSV)	Crayfish infected with WSSV displayed decreased intestinal microbiota diversity and richness and relative abundance of an opportunistic pathogen (*Aeromonas*) increased. The potential pathogenicity in the gut microbiota of WSSV-infected crayfish was increased compared to healthy controls.	Xue et al. (2022)
Black tiger shrimp (*Penaeus monodon*)	Hepatopancreas	Vibriosis	Infected and non-infected shrimp obtained from six hatcheries. Alpha diversity was reduced in the hepatopancreas of infected shrimp. Eight bacterial genera were associated with a shift in the microbiome in infected shrimp.	Foysal et al. (2021)
Pacific oyster (*Crassostrea gigas*)	Whole oyster	Pacific oyster mortality syndrome (POMS)	Oysters from resistant or susceptible families infected with POMS. All individuals from susceptible families died, but during early infection, microbiota of the whole oyster showed a reduction in evenness compared to resistant families.	Clerissi et al. (2020)
Atlantic salmon (*Salmo salar*)	Gill	Amoebic gill disease (AGD)	The microbiota of AGD-affected and non-affected gill tissue biopsies was compared. Bacterial diversity was significantly reduced in biopsies from both AGD-affected and un-affected gill tissue from infected fish compared to uninfected fish. Lesions in AGD-affected tissue contained higher abundance of *Tenacibaculum dicentrarchi*.	Slinger et al. (2020)
Atlantic salmon (*Salmo salar*)	Mouth	Tenacibaculosis (Yellow Mouth, YM)	Comparing YM infected fish with uninfected control, a reduction in microbiota diversity and distinct dysbiosis were identified. High levels of the primary causative agent *Tenacibaculum maritimum* were seen in infected and healthy fish indicating additional unknown factors responsible for pathology of YM. An association between *T. maritimum* and *Vibrio* abudance was identified.	Wynne et al. (2020)
Red abalone (*Haliotis rufescens*)	Digestive gland	Withering syndrome (WS)	In red abalone affected by WS, *Candidatus Xenohaliotis californiensis* replaced *Mycoplasma* as the dominant taxon in the digestive gland microbiota*. C. X. californiensis* was also sequenced in healthy specimens, suggesting that the ratio between the two taxa may be more important in determining pathogenicity of WS.	Villasante et al. (2020)
Yellowtail Kingfish (*Seriola lalandi*)	Gut and skin	Gut enteritis	Microbiota and gene expression in gut and skin analyzed in fish affected by gut inflammation. The gut microbiota of affected fish was dominated by a *Mycoplasmataceae* sp., while a reduction in microbial diversity in the skin was identified at the early stages of disease. Gene expression analysis revealed little differentiation in the gut between healthy and affected fish while extensive differences were identified in the skin, related to pathways indicative of a weakened host.	Legrand et al. (2020a)
Pacific whiteleg shrimp (*Litopenaeus vannamei*)	Gut	White spot syndrome virus (WSSV)	In shrimp infected with WSSV, there was no significant impact on bacterial richness or diversity. However, community composition was different between infected and non-infected individuals with increased *Proteobacteria* and *Fusobacteria*, but reduced *Bacteroidetes* and *Tenericutes* in infected shrimp.	Wang et al. (2019)

### 4.1. Can a “healthy” microbiome be defined for aquaculture practices?

Attempts have been made in the past to define a “healthy” microbiome in terms of aquaculture but realistically a single consistent “healthy” microbiome is unlikely to exist, even within a single species in a single culture system (Infante-Villamil et al., 2021; Rajeev et al., 2021). Thus, perhaps a desirable microbiome for aquaculture should instead be defined as one that has the capacity to adjust to its environment, and in doing so maintain a beneficial symbiotic relationship with the host. Maintenance of community homeostasis historically has been considered important, but this should be expanded to considering community homeostasis in terms of functionality rather than taxonomic identity. Microbial communities can be remarkably dynamic in response to changes in their environment and a highly plastic, dynamic microbiome may also contribute to host phenotypic plasticity, and thus host ability to tolerate environmental stressors. The “Anna Karenina principle” (Zaneveld et al., 2017) infers that stressors create more stochastic microbiomes as a consequence of a dampening of the host’s ability to regulate its microbial communities, resulting in increased inter-individual variation in microbiomes of hosts experiencing stress (Boutin et al., 2013). However, the contrasting pattern has also been observed, with remarkably similar microbiome responses to environmental disturbance which have been attributed to reduced competition allowing increased dominance of certain taxa (Webster et al., 2019). Such differences may well reflect differences in external environment. In open systems with more variation in environmental microbes, we might expect greater stochasticity, but in closed systems with less environmental microbial variation, we might expect to see the domination of certain taxa.

Although maintaining low levels of stress in aquaculture is a central tenet of stock management, even routine husbandry practices can induce stress, which may increase disease susceptibility (Eissa and Wang, 2016). A microbiome with a high adaptive plasticity is less likely to be disturbed by environmental stressors and therefore there are less likely to be knock-on adverse effects of microbiome disturbance on host health. A plastic microbiome may also confer specific fitness benefits to the host (e.g., an ability to metabolize or sequester dietary toxins, adapt to novel nutrient or feed sources, or to cope with changing water temperatures) (Alberdi et al., 2016; Macke et al., 2017). It is important to consider that the nature and outcomes of stress responses will depend on the nature of the existing microbial community, with different mucosal sites displaying differing responses to stress in terms of both cortisol levels and microbiome dynamics (Webster et al., 2019). Despite variable responses, identification of markers of stress or dysbiosis across datasets could enable these to be leveraged in real-time microbiome monitoring in aquaculture through non-invasive sampling of water, feces, or external mucus to detect microbial signatures of potential ill-health. Going a step further, the future ideal would be to actively intervene and manipulate the microbiome during these periods of dysbiosis to help re-balance disruptions and maintain optimal host health.

### 4.2. Dysbiosis and rebiosis—an opportunity to condition microbiomes for sustainable aquaculture?

As explained above, the first microbial colonizers are key in determining succession via competitive exclusion or “colonization resistance,” competing for nutrients and physical space (Vonaesch et al., 2018). Post-colonization, development of the fish microbiome is characterized by periods of establishment and proliferation which are especially dynamic and strongly influenced by environment, and periods of stabilization which are strongly influenced by the host. Husbandry practices can introduce instability to the environment and these transitional periods are often accompanied by high levels of mortality or increased disease susceptibility (Paul-Pont et al., 2013; Johansson et al., 2016). As an example, on-growing of Atlantic salmon requires transition from a freshwater to a saltwater environment, exposing commensal microbes to a distinct shift in salinity and ultimately creating a new niche for salt-tolerant taxa to the detriment of freshwater commensals. Such distinct shifts in environmental parameters are accompanied by rapid remodeling of host microbiomes (Dehler et al., 2017; Lorgen-Ritchie et al., 2021) characterized by an initial loss of community structure, a process known as dysbiosis, followed by re-establishment of a new community—rebiosis. Periods of rapid change in the microbiome are especially sensitive to external influences of incoming microbes that may later be more inhibited by colonization resistance, and this may have a lasting effect on microbiome composition and function via priority effects (Debray et al., 2021). This could have important implications for aquaculture as these periods may be especially sensitive to disruption but may also represent an opportunity for increased sensitivity to conditioning.

Dysbiosis in the naturally occurring microbiome of the host often coincides with infection or poor health, from invertebrates to humans. Dysbiotic states may manifest as (i) loss of beneficial organisms, (ii) expansion of pathobionts and/or (iii) loss of microbial diversity (Petersen and Round, 2014). It is important to consider community interactions when trying to understand these processes. Ecological theory suggests diverse communities are more resistant to dysbiosis and invasion by opportunistic pathogens (Levine and D'Antonio, 1999) as they have more diverse functionality and more effective competitive exclusion. Salmon experimentally infected with the copepod “louse” ectoparasite, *Lepeophtherius salmonis,* have been shown to display a reduced bacterial richness in their skin microbiome (Llewellyn et al., 2017). A similar pattern has been observed in the gut of shrimp suffering white feces syndrome (Hou et al., 2018). However, in shrimp suffering from white spot syndrome virus (WSSV), no significant difference in diversity was observed although community composition was distinct in control vs. diseased animals (Wang et al., 2019). Moving forward, defining dysbiosis in terms of loss of functional integrity and knock-on effects on host health may be more useful when it comes to potential development of therapeutics.

It remains unclear whether dysbiosis occurs in response to infection or if opportunists exploit an existing or incipient dysbiotic state, perhaps induced by change in environmental conditions or host physiology or immune capabilities, in order to infect. A combination of both is probably most likely—infection may well exacerbate an already dysbiotic state. To illustrate this scenario, production of the Pacific oyster (*C. gigas*) is hampered by disease, especially Pacific oyster mortality syndrome (POMS) that has caused major economic losses (Segarra et al., 2010). POMS is a polymicrobial disease where oysters become infected by osterid herpes virus 1 μvar (OSHV-1 μvar) and this leads to immune suppression facilitating dysbiotic shifts in the oyster’s microbiome. This altered microbiome, the pathobiome, is characterized by dominance in taxa including *Vibrio*, *Aliivibrio*, *Arcobacter*, *Marinobacterium*, *Marinomonas*, *Photobacterium*, *Psychrobium*, and *Pschromonas* (de Lorgeril et al., 2018; Clerissi et al., 2020; Petton et al., 2021; Richard et al., 2021). Affected oysters show bacteremia where opportunistic vibrios including *V. aestuarianus* cause significant mortality. Oyster families displaying greater microbial diversity within individuals tend to be less likely to suffer from POMS (Clerissi et al., 2020) and selective breeding programs may improve disease resistance and hence reduce the impact of this condition (Dégremont et al., 2020). Similarly in Atlantic salmon, amoebic gill disease (AGD) is a increasing gill health issue, the etiological agent of which is the free-living amoeba *Neoparamoeba perurans* (Crosbie et al., 2012). AGD infection is initiated by adherence of *N. perurans* to the gill mucosa where commensal bacteria persist (Embar-Gopinath et al., 2005). Microbial profiling revealed lower bacterial diversity and a moderate positive correlation between *N. perurans* and *Tenacibaculum dicentarchi* in infected tissues (Slinger et al., 2020). Altering bacterial load on the gills prior to *N. perurans* infection using antimicrobial treatments resulted in differing progression of AGD with significantly higher severity observed in chloramine-trihydrate (CI-T) treated fish vs. control fish. High levels of *Tenacibaculum* were observed in the CI-T group again suggesting a protagonistic role for this taxon in AGD infection highlighting the potential for dysbiosis to allow expansion of potentially pathogenic communities which may affect subsequent disease progression (Slinger et al., 2021). Future research is needed to identify useful commonalities in these intertwined relationships between dysbiosis, disease progression, and host health.

## 5. Moving from microbiome characterization to beneficial manipulation in aquaculture

Identifying commensal microbes and their dynamics is the first step in understanding the value of microbiomes for aquaculture productivity and sustainability. If “*the microbiome*” (accepting there are many), is to be used as a tool to improve health and performance of the animals, we need to know not just which microbes are present, but how these microbial communities function (Gibbons, 2017). In recent years there has been a distinct shift in microbiome research to “function over phylogeny.” Functionality has historically been inferred from high-throughput amplicon sequencing, but predictions are limited in their use in aquaculture studies due to poor taxonomic resolution and low numbers of characterized bacterial genomes across aquaculture environments (Infante-Villamil et al., 2021). Additionally, such predictions relate to a specific set of conditions, and functional analysis tools assume that all genes are transcriptionally active in all microbial taxa at that moment in time, which more often than not is simply not the case. More holistic ‘omics strategies such as metagenomics (Tepaamorndech et al., 2020), metatranscriptomics (Nam et al., 2018), metaproteomics (Jose et al., 2020), and metabolomics (Roques et al., 2020), as well as integration of these approaches (multi-omics) (Uengwetwanit et al., 2020) are increasingly being adopted. A step further is to consider the “hologenome,” that is the collection of genes in the microbiomes along with those of the host (Rosenberg et al., 2007; Rosenberg and Zilber-Rosenberg, 2018). The strength of these approaches is the ability to examine microbiomes in terms of community ecology, considering interactions among microbiota, with the host and with the environment, but integration requires well designed and executed sampling strategies, sufficient levels of sampling (generally producing very large datasets), and complex bioinformatics analyses. Omics approaches are key to uncovering contrasts and commonalities between microbiome function across aquaculture but must be applied in combination with carefully designed mechanistic studies.

### 5.1. Modeling a desirable microbiome function for aquaculture

One approach to understanding functionality is to use germ-free (GF) or gnotobiotic models. Gnotobiotic refers to animals hatched and reared in a sterile environment which provide a “blank canvas” on which to test the impact of introducing a single microbe or a community of microbes. Although gnotobiotic research has been applied to aquaculture species since the 1940s (Zhang et al., 2020), full use of this approach as a tool to understand microbiome functionality is only now being realized. Nile tilapia (*Oreochromis niloticus*) models have been used to test the efficacy of therapeutics and to study colonization by free-living microbes (Situmorang et al., 2014; Giatsis et al., 2016) while in gnotobiotic rainbow trout larvae commensal taxa which offered protection against pathogenic infection have been identified (Pérez-Pascual et al., 2021). Furthermore, cod (*Gadus morhua*) models have recently been used to study the role of microbiota in innate immune development and gut morphology (Vestrum et al., 2022). Gnotobiotic oyster (*Crassostrea gigas*) models have also been utilized to detect effects of pathogens (Arzul et al., 2001) while in crustacea, germ-free *Artemia* brine-shrimp (Baruah et al., 2017) and *Daphnia* (Macke et al., 2020) models are widely used to study host-microbiome-environment interactions.

Pioneering work in zebrafish (reviewed in Murdoch and Rawls, 2019) established a germ-free model, but also a conventionalized model by harvesting bacteria from tank water of conventionally reared zebrafish and adding this community to gnotobiotic zebrafish medium to colonize germ-free animals (Rawls et al., 2004). Using these models and transcriptomic analysis, 212 genes regulated by the microbiota were identified in the intestine, and 59 responses were conserved in the mouse intestine indicative of an evolutionary conserved role of the vertebrate gut microbiome. Similarly, exposure of germ free zebrafish to yeast in the form of *Pseudozyma* sp. elucidated differential expression of 59 genes compared to naive controls, many involved in metabolism and immune response, indicating that commensal fungi also have the potential to influence the early development of fish larvae (Siriyappagouder et al., 2020). This work was extended using reciprocal gut microbiome transplants from zebrafish and mice to germ-free recipients and identified the importance of host habitat selection (Rawls et al., 2006). Transplanted communities resembled the community of origin in terms of taxonomic lineages present, but relative abundances were modified to resemble the normal microbial community composition of the recipient host. These transplantation studies are important in understanding microbiome function and selection pressures during colonization, but also have the potential to be adapted for therapeutic means similar to fecal microbiota transplantation (FMT) (Petersen and Round, 2014; Vargas-Albores et al., 2021). Such approaches may well be used in aquaculture as understanding of functionality of microbiomes improves.

Limitations exist in terms of long-term germ-free husbandry and untangling complexities of microbe-microbe interactions. Genetic manipulations including knock-out, knock-in and clonal approaches are additional avenues by which to uncover mechanistic microbiome pathways and potential genetic influences on microbiome composition. IgT, a teleost mucosal immunoglobulin believed to be an important adaptive immune determinant of microbial homeostasis, was depleted in a rainbow trout model. This depletion resulted in dysbiosis characterized by an increased presence of pathobionts, tissue damage and inflammation as well as increased susceptibility to mucosal parasites, highlighting the importance of this immunoglobulin in microbiome homeostasis in finfish (Xu et al., 2020). Clonal lines of aquaculture species are an additional tool that can be utilized to uncover genetic contributions to microbiome composition under varying environmental conditions. Clonal lines have advantages over inbred lines by avoiding artificially low levels of heterozygosity and inbreeding depression. The first clonal line of Atlantic salmon has recently been verified (Hansen et al., 2020) adding to previous lines in aquaculture species, and future work could potentially see the extension of aquaculture breeding programs to select for resistant microbiome types if these can be determined. These tools take time to develop, but with the increasing availability of fully sequenced genomes of aquaculture species, genetic manipulations to enhance functional understanding are becoming more accessible. Despite the evolution of ‘omic tools, the practice of culturing microbes is also making a resurgence as a tool for understanding functionality by enabling mechanistic experiments to determine physiological processes of microbes, or how they interact with their host. Culturing also allows for more in depth genomic analyses of taxa and discovery and description of novel taxa which can then feed into sequencing databases (Hitch et al., 2021).

### 5.2. Applying microbial manipulation effectively in aquaculture systems

Potential therapeutics for the aquaculture industry must be biologically- and cost-effective to manufacture and apply. Evidence-based procedures should be adopted with a view to identifying pivotal taxa, whether that is a single species which plays a role in a specific disease or resistance to infection provided by controlled communities of bacteria and other microbes.

Gut microbiomes of many organisms play an important role in aiding digestion and supporting gut (and organismal) health. Perhaps the most well-known or widely-applied example of microbial therapeutics in aquaculture (and human) health are the inclusion of pre- pro- or synbiotics in the diet. Probiotics refer to live microbial feed supplements which promote host health (Hill et al., 2014) whereas pre-biotics are non-digestible feed ingredients which selectively stimulate the growth of one or a limited number of particular microbes (Yadav et al., 2022). Synbiotics refers to products containing both pre- and pro-biotics working in synergy (Schrezenmeir and de Vrese, 2001). Recent reviews on prebiotic and probiotic use have shown favorable outcomes including increased growth or survival, improved immune responses, increased digestive efficiency, and improved water quality (López Nadal et al., 2020; El-Saadony et al., 2021; Knipe et al., 2021), but caution that more knowledge is needed in fish to promote efficacy (Brugman et al., 2018).

Probiotics can exert their function via the principles of competitive exclusion, be that exploitation or interference competition (Knipe et al., 2021). In exploitation, competition is indirect and characterized by consumption of resources while in interference, direct harm is caused, for example via the production of antimicrobial compounds. For example, the lactic acid bacteria (LAB) family of probiotics produce antimicrobial substances including lactic acid, acetic acid, hydrogen peroxide and bacteriocins interfering with the growth of other bacteria (Ringø, 2020; Ringø et al., 2020). Feeding shrimp (*Litopenaeus stylirostris*) with a diet containing the probiotic *Pediococcus acidilactici* resulted in the purging of potentially pathogenic vibrios from the gut (Castex et al., 2008). Similarly, in freshwater marron crayfish (*Cherax cainii*), feeding with *Clostridium butyricum* resulted in a reduction of *Vibrio* and *Aeromonas* counts in the hindgut (Foysal et al., 2019). In Atlantic salmon, feeding with *Peiococcus acidilactici* resulted in distinct reduction of *Fusobacteriia*, *Clostridiales*, *Actinomycetales*, *Pasteurellales*, and *Streptococcacea*, and an increase in *Bradyrhizobiaceae* in gut mucosa, but also identified that this probiotic effect is dependent on the water habitat (fresh vs. seawater) (Jaramillo-Torres et al., 2019). Contrastingly, in Nile tilapia, probiotic feeding with a mix of three *Bacillus* species did not modify gut microbiota to any large extent (Adeoye et al., 2016). Despite differences in efficacy, intervention with probiotics, prebiotics or synbiotics in an aquaculture setting more often than not illicit changes in the host microbiome (Table 3).The complex and dynamic nature of changing environments in aquaculture systems has confounded wide usage of probiotics to date. Variability in efficacy may be a consequence of therapeutics often being derived from non-aquatic hosts or systems, resulting in poor tolerance of biophysical conditions within the host and subsequently poor survival and establishment. In reality, to ensure efficacy, any potential pro- or pre-biotic therapeutic needs to be evaluated under different environmental circumstances to target optimum windows of application for maximum impact, particularly in aquaculture where the environment itself can often be manipulated. Although pre- and pro-biotics are generally applied via feed, the opportunity also exists in aquaculture systems to apply such therapeutics directly to the water with a view to directly influencing external microbial communities (Rowley, 2022). Utilizing specific microbial probiotics targeted at specific pathogens carries a degree of risk whether it be the invasion of secondary pathogens or evolution of target pathogen to evade the mechanism of exclusion (Knipe et al., 2021). AMPs are an intriguing route toward developing therapeutic compounds in aquaculture as they have the power to modulate the immune system while maintaining a low probability of the development of bacterial resistance (Chaturvedi et al., 2020). The gut microbiome represents an environment which may harbor AMPs with therapeutic and microbiome sequencing datasets can be mined to uncover candidate AMPs (Dong et al., 2017).

**Table 3:** Key published literature analyzing the impact of probiotic, prebiotic, and synbiotic interventions on microbiome composition.

Species	Intervention (s)	Supplement (s)	Description	References
Rainbow trout (*Oncorhynchus mykiss*)	Probiotic and synbiotic	Probiotic – BACTOCELL (*Pediococcus acidilactici*) Synbiotic – BACTOCELL + galacto-oligosaccharides	Multiomics approach identified changes in the microbiome with a reduced relative abundance of *Candidatus Mycoplasma salmoninae* directly associated with changes in microbial arginine biosynthesis and terpenoid backbone synthesis pathways. Additionally, differences in microbiota composition were associated with alterations in metabolomic profiles.	Rasmussen et al. (2022)
Nile tilapia (*Oreochromis niloticus*)	Prebiotic	Honey	Oligosaccharides of honey can act as a prebiotic. The impact of four dietary treatments (honey inclusion of 0, 0.25, 0.5 and 1%) were tested. Honey inclusion increased growth performance, reduced feed conversion ratio, increased microvilli length, and improved microbiota diversity in the gut of tilapia with the best dietary dose determined to be 1%.	Aryati et al. (2021)
Pacific whiteleg shrimp (*Litopenaeus vannamei*)	Probiotic, prebiotic and synbiotic	Probiotic – *Bacillus* sp. NP_5_ Rf^R^ Prebiotic – honey Synbiotic – honey + *Bacillus* sp. NP_5_ Rf^R^	Shrimp were fed with control, pre-, pro- or symbiotic treatments. All experimental treatments resulted in increased growth rate, feed conversion ratios, and digestive enzyme activities compared to controls with the prebiotic treatment being the most effective. The prebiotic also increased the presence of a number of known probiotic candidates.	Hasyimi et al. (2020)
Pacific whiteleg shrimp (*Litopenaeus vannamei*)	Prebiotic	Mannan oligosaccharides	Shrimp in intensive pond culture were fed with mannan oligosaccharide (MOS) supplementation. Shrimp survival was increased by 30%. Changes were identified in the gut microbiota with the prevalence of potential opportunistic pathogens (*Vibrio*, *Aeromonas*, *Shewanella*) negligible in MOS fed shrimp.	Gainza and Romero (2020)
Atlantic salmon (*Salmo salar*)	Probiotic	*Pediococcus acidilactici* MA18/5 M	The impact of seawater transfer (SWT) on gut microbiome in fish with or without dietary inclusion of probiotic was assessed. Both probiotic supplementation and SWT impacted gut microbiome composition. A higher antiviral response of fish fed the probiotic diet was indicative of a causal link between microbiome composition and activation of the antiviral response.	Jaramillo-Torres et al. (2019)
Marron crayfish (*Cherax cainii*)	Probiotic	*Clostridium butyricum*	Feeding with probiotic supplementation resulted in increased growth, attributed to an increase in molt occurrence. Probiotic inclusion also increased bacterial diversity and abundance of pathogenic taxa (*Vibrio* and *Aeromonas*) were significantly reduced. Additionally, the expression of immune-responsive genes was modulated in probiotic fed individuals when challenged with *Vibrio mimicus*.	Foysal et al. (2019)
Eastern oyster (*Crassostrea virginica*)	Probiotic	*Bacillus pumilus* RI06-95	Daily addition of a probiotic to culture water had no impact on the diversity of bacterial communities in oyster larvae and water. However, abundances of *Oceanospirillales* and *Bacillus* were higher in probiotic treated water and oyster larvae, and co-occurrence network analysis indicated a role for probiotic treatment in decreasing potentially pathogenic taxa.	Stevick et al. (2019)

## 6. Future prospects

The importance of the microbiome in the future development of sustainable aquaculture systems is clearly evident considering the vast outpouring of research in this area over the past 5–10 years, including a raft of recent reviews (de Bruijn et al., 2018; Perry et al., 2020; Legrand et al., 2020b; Infante-Villamil et al., 2021; Parata et al., 2021; Rajeev et al., 2021; Vargas-Albores et al., 2021; Diwan et al., 2022; Paillard et al., 2022). Despite this great interest, few focused studies have been conducted relating to the impact of microbiomes on host health and subsequent productivity, and the vast majority of studies have focused only on bacteria, mainly due to technical difficulties or cost associated with characterizing eukaryotes and viruses. Understanding more about these other communities and their interactions is imperative and also holds many opportunities for microbiome engineering, for example using phage to control opportunistic pathogens (Donati et al., 2021). There is now a critical need for better understanding of microbiome function in the context of aquaculture for enabling application to enhancing improved production systems. Poor animal welfare and loss of revenue due to disease remains one of the most restrictive issues in aquaculture development. One future focus should be pinpointing optimum periods for microbiome conditioning or manipulation, both during immune system development at larval stages and within periods of dysbiosis caused by disease or husbandry practices. Directing studies also toward functionality over identity will also be key in unlocking the full therapeutic potential of the microbiome in the aquaculture sector and employing next generation sequencing technologies in tandem with culture-based approaches will be essential in making this transition.

## Author contributions

SM, CT, AR, and ML-R conceived the original concept. TW, JM, and DB expanded on scientific areas. JM and ML-R created the figures. ML-R wrote the initial draft and managed. All authors edited and contributed equally to the final manuscript.

## Funding

This work was supported by BBSRC grants BB/P017223/1 and BB/P017215/1 for the Aquaculture Research Hub (ARCH-UK).

## Conflict of interest

The authors declare that the research was conducted in the absence of any commercial or financial relationships that could be construed as a potential conflict of interest.

## Publisher’s note

All claims expressed in this article are solely those of the authors and do not necessarily represent those of their affiliated organizations, or those of the publisher, the editors and the reviewers. Any product that may be evaluated in this article, or claim that may be made by its manufacturer, is not guaranteed or endorsed by the publisher.
